# Acute necrotizing pancreatitis

**DOI:** 10.4322/acr.2020.215

**Published:** 2020-11-20

**Authors:** Deepika Phogat, Mukul Bajpai, Pranshu Agrawal, Prosenjit Ganguli

**Affiliations:** 1 151 Base Hospital, Department of Lab Sciences, Guwahati, Assam, India; 2 Command Hospital Eastern Command, Department of Lab Sciences, Kolkata, West Bengal, India

Acute pancreatitis (AP) is an inflammatory condition of the pancreas that results from inappropriate intrapancreatic activation of enzymes like trypsin with downstream activation of other proenzymes into the interstitial space (rather than the acinar lumen) that causes the autodigestion of the pancreatic parenchyma, fat tissue and damages elastic fibres of blood vessels along with a marked inflammatory response.^1^ The most common etiological factors are alcohol misuse, cholelithiasis, drugs, trauma, hyperlipidemia and post retrograde cholangiopancreatography.^1^ ^,^ ^2^ Smoking and diabetes mellitus seem to increase the risk for acute pancreatitis.^3^ ^,^ ^4^ The incidence of AP ranges between 13 to 45 per 100,000 people.^5^ Chronic alcohol consumption results in the secretion of protein-rich pancreatic fluid leading to the deposition of protein plugs and obstruction of the pancreatic ducts besides the direct effects of alcohol on the pancreatic acinar and stellate pancreatic cells. In the biliary pancreatitis, the toxic effect of bile acids within the acinar cells increase intra-acinar calcium concentration triggering the inflammatory response.^1^ ^,^ ^6^. Histologically, acute pancreatitis is classified as interstitial edematous and necrotizing subtypes.^7^ The necrotizing form is associated with high mortality.^8^ Among the grading scores of the severity of acute pancreatitis, like the Ranson Score (that is based on the clinical and lab investigation parameters), those based upon the computed tomographic imaging findings, like the Revised Atlanta Classification Score are more accurate with >90%accuracy.^7^ ^,^ ^9^

**Figure 1:**
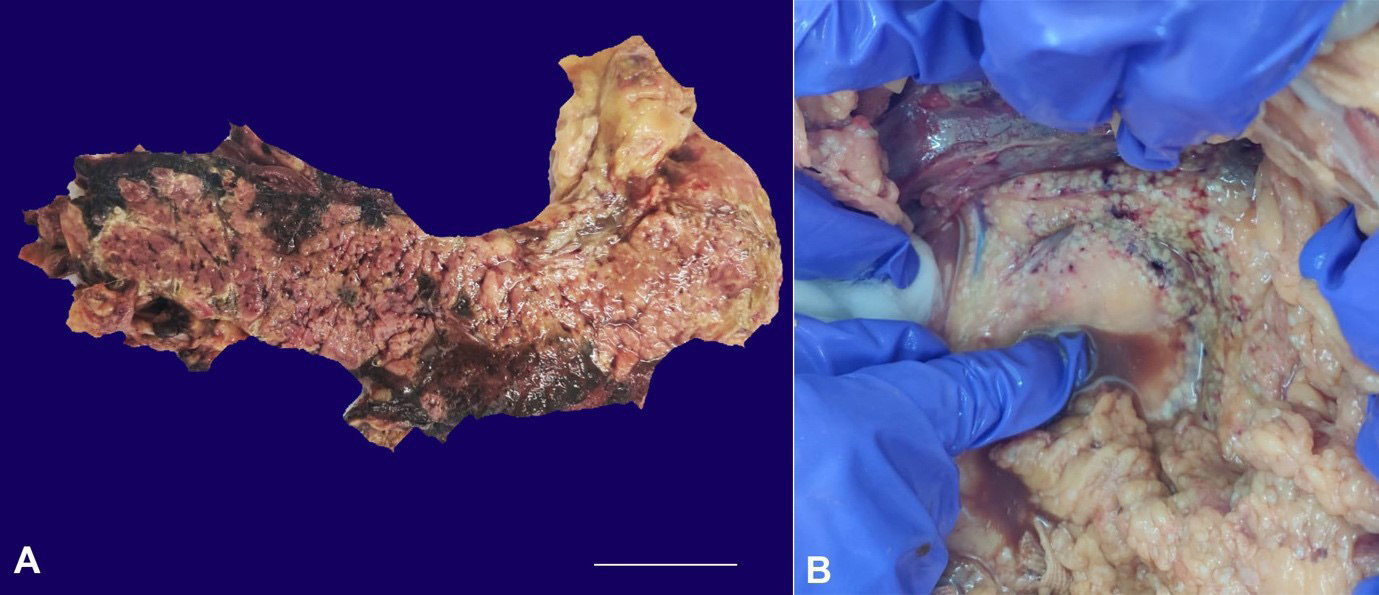
Gross view of the pancreas and peritoneal fat tissue. **A** – Cut surface of pancreas showing extensive parenchymal necrosis accompanied by hemorrhage within the gland; **B** – Foci of fat necrosis in the form of small white chalky deposits in the peritoneal cavity along brown tinged fluid with globules of fat.

The above picture refers to the case of a 33-year-old male, known alcohol abuser, presented with sudden onset acute epigastric pain over the last six hours. His laboratory investigations revealed serum lipase level of 3412 U/L (RR:73-393IU/L); serum amylase level of 358 IU/L(RR: 85-115IU/L), serum calcium of 6.1 mg/dl (RR:8.5-10mg/dl), blood urea of 66mg/dL (RR;10-50mg/dl) and creatinine of 2.5mg/dL (RR;0.8-1.0mg/dl). The abdominal computed tomography revealed features of acute pancreatitis with the Revised Atlanta Classification score of 8/10. Despite aggressive management, the patient developed features of Systemic Inflammatory Response Syndrome and succumbed to his condition. On Autopsy, the pancreas was grossly enlarged and edematous with loss of surface lobulations, weighing 500gm (mean RR; 110 g). The cut surface showed extensive parenchymal necrosis accompanied by hemorrhage intermingling the substance of the gland (Figure 1A). Foci of fat necrosis in the form of small white chalky deposits were present in the peritoneal cavity adjacent to the pancreas (Figure 1B). Histopathological examination revealed areas of parenchymal necrosis surrounded by foci of shadowy outlines of necrotic fat cells with basophilic calcium deposits. Microbiological analysis of the necrotic pancreatic tissue did not reveal the presence of any bacterial organisms.

Severe acute pancreatitis is associated with an overall mortality of 30%, with 5% deaths occurring within the first week of illness.^10^ ^,^ ^11^ The laboratory findings include marked elevation of serum amylase levels during the first 24 hours, followed by a rising serum lipase level by 72 to 96 hours after the beginning of the attack. Hypocalcemia may occur from precipitation of calcium soaps in necrotic fat. Direct visualization of enlarged, inflamed pancreas by CT scanning is useful.

The key to the acute pancreatitis management is resting the pancreas by total restriction of oral intake and by supportive therapy with intravenous fluids and analgesics. Acute respiratory distress syndrome and acute renal tubular necrosis are ominous complications. In 40-60%of patients with acute necrotizing pancreatitis, the necrotic debris becomes infected, usually by gram-negative organisms from the alimentary tract, further complicating the clinical course. Infected Necrosis has a mortality of 100%.^12^ ^,^ ^13^ Systemic organ failure and necrosis in the pancreas are both poor prognostic findings.
